# Safety and Efficacy of Tirofiban in Rescue Treatment for Acute Intracranial Intraprocedural Stent Thrombosis

**DOI:** 10.3389/fneur.2020.00492

**Published:** 2020-06-16

**Authors:** Lili Sun, Jinping Zhang, Yun Song, Wei Zhao, Meimei Zheng, Jun Zhang, Hao Yin, Wei Wang, Yao Meng, Jiyou Tang, Ju Han

**Affiliations:** ^1^Department of Neurology, Shandong Qianfoshan Hospital, Cheeloo College of Medicine, Shandong University, Jinan, China; ^2^Department of Neurology, The First Affiliated Hospital of Shandong First Medical University, Jinan, China

**Keywords:** stent thrombosis, stroke, intracranial atherosclerotic stenosis, rescue treatment, glycoprotein IIb/IIIa inhibitor, tirofiban, outcome

## Abstract

**Background and Purpose:** The incidence of acute intraprocedural stent thrombosis (AIST) during stenting of intracranial atherosclerotic stenosis (ICAS) has seldom been reported and evidence regarding the treatment of AIST is lacking. We aim to investigate the incidence of AIST during stenting of ICAS in our institute, assess the preliminary efficacy and safety of rescue treatment of tirofiban for these patients.

**Methods:** From September 2016 to May 2019, all symptomatic ICAS patients who underwent intracranial stenting in our institute were prospectively registered into this study, of which patients with AIST were retrospectively reviewed to extract baseline characteristics, perioperative management, procedural details, angiographic, and clinical outcomes. Rescue treatment of tirofiban for AIST was assessed by recanalization of the culprit vessel and periprocedural death, hemorrhage, and ischemic stroke.

**Results:** Acute intraprocedural stent thrombosis developed in 12 (6.2%) patients within 30 min after stent placement of 194 patients. All 12 cases were successfully recanalized with modified Treatment in Cerebral Ischemia (mTICI) 3 and Arterial Occlusive Lesion (AOL) 3 after rescue treatment of tirofiban alone. There was no perioperative death or any hemorrhagic complication. Three patients suffered perioperative ischemic stroke.

**Conclusions:** We observed a non-negligible rate of AIST during intracranial stenting procedures for ICAS. Intra-arterial bolus followed by intravenous tirofiban infusion seems to be efficacious and safe for AIST during stent placement for ICAS, without increasing the rate of hemorrhagic complications and death.

## Introduction

Acute intraprocedural stent thrombosis (AIST) is a challenging and usually poor prognostic event during stenting of intracranial atherosclerotic stenosis (ICAS), frequently associated with large cerebral infarction or death (1). The reported incidence was as high as 14.6% previously during the Wingspan intracranial stent placement (2) and much higher during stent placement in middle cerebral artery (3). However, there is no clear consensus or evidence-based protocol for the management of this potentially devastating periprocedural complication. Taking all the potential danger of intracranial stenting procedures into account, it is important to gain deeper insight into AIST to prevent and to treat this complication.

The use of glycoprotein IIb/IIIa (Gp IIb/IIIa) inhibitor abciximab or tirofiban as salvage therapy for AIST during intracranial stenting has been described in certain clinical scenarios, which suggests that GpIIb/IIIa inhibitor might be beneficial and safe (1–3). Here, in this report, we retrospectively review consecutive patients who underwent stent placement for ICAS in our institute and identified all patients with AIST. The incidence of AIST was investigated and our experiences of tirofiban injected as a rescue therapy in AIST during stent placement for ICAS was presented. We hypothesized that intra-arterial bolus of tirofiban followed by intravenous maintenance was sufficient to resolve AIST with a low risk of hemorrhagic complications and death.

## Materials and Methods

### Study Population

We retrospectively evaluated clinical and radiological data of all patients with ICAS who underwent stenting between September 2016 and May 2019 at our institute. The inclusion criteria for elective intracranial stenting were as follows: (a) intracranial atherosclerosis as the primary etiology, other potential causes of stenosis, such as Moyamoya syndrome, vasculitis, or arterial dissection were excluded; (b) severe intracranial atherosclerotic stenosis ≥ 70% as defined by the WASID trial (Warfarin Vs. Aspirin for Symptomatic Intracranial Disease) (4); (c) recurrent ischemic stroke or transient ischemic attack attributable to the culprit artery, despite being on dual-antiplatelet therapy, statin, and vascular risk factor control; and (d) cerebral hypoperfusion in the territory supplied by the culprit artery, which was supposed to be on the basis of clinical and imaging evidence. The exclusion criteria were as follows: (a) patients with severe ICAS and aneurysm were treated at the same time; (b) patients with abnormal blood platelets and coagulopathy. Data on patient demographics, lesion location, stenosis degree, stent characteristics (stent length and diameter), and the main types of stents were collected. Cases where AIST was encountered were selected for the study.

Informed consent was obtained from all patients or their legal representatives after detailed explanation of the procedures. The protocol was approved by the central ethics committee at Shandong Qianfoshan Hospital. This study was approved by the institutional review board of Shandong Qianfoshan Hospital, Shandong University. Given its retrospective nature, the study does not require registration.

### Perioperative Management and Stenting Procedure

All patients were on dual antiplatelet agents (100 mg aspirin and 75 mg clopidogrel) daily for at least 5 days before stenting. Antiplatelet resistance test was considered to guide antiplatelet drug treatment. In our center, we would increase the dose of aspirin or clopidogrel if inadequate inhibition was found. Cilostazol and ticagrelor may be other options. The procedures were performed by an experienced neurointerventionist, who had done at least 100 endovascular procedures for ICAS.

All stents were performed under general anesthesia. Intravenous heparin was administered during the procedure to maintain the activated coagulation time between 250 and 300 s. Device selection depended on arterial access, lesion morphology, and vascular characteristics. The basic principles were as follows: (a) For lesions <10 mm, with straightforward arterial access, the balloon-mounted stent (Apollo, MicroPort, Shanghai, China) was chosen. (b) For lesions >10 mm with tortuous arterial access, or lesions with a significant mismatch in the diameter between the proximal and distal segment, the gateway balloon plus Wingspan stent system (Boston Scientific, Massachusetts, USA) or Neuroform EZ stent (Boston Scientific, Massachusetts, USA) was preferred.

Intraprocedural angiography was performed at about 10-min intervals for at least 40 min. When shadow defects were observed within the stent on intraprocedural angiograms, AIST was recorded. Its onset was measured from the time of stent placement. The angiographic confirmation of AIST was the presence of a thrombus that originated in the stent or within 5 mm of the stent edge. Two experimented neurointerventionists reviewed the angiographic images before and after the rescue treatment. AIST was graded according to the following scale (2): (a) grade 1: visible thrombus without any flow defects, (b) grade 2: visible and flow-limiting thrombosis, (c) grade 3: complete stent occlusion. The time of thrombosis diagnosis and disappearance was noted.

Patients with AIST grade 1–2 were given tirofiban (Hengkang, Lunan Better Pharmaceutical Co., Ltd., Shandong, China) through the guiding catheter, the initial intra-arterial administration dose was 10 μg/kg according to a body weight–adjusted dosage regimen and the injection was completed within 3 min. For treatment of AIST grade 3, a Synchro micro-guidewire (Stryker Neurovascular, Utah, USA), assisted by an Excelsior SL-10 soft micro-catheter (Stryker Neurovascular, Cork, Ireland), was introduced into the occluded culprit artery and carefully passed through the occluded lesion to the distal segment. After withdrawing the micro-guidewire, tirofiban (10 μg/kg) was injection through the microcatheter to the occluded lesion segment, the distal and the proximal segment of the stent. The distal, middle, and proximal parts of the thrombus were each injected with 1/3 of the dose. If a remnant thrombus was identified via angiography image, an additional 10 μg/kg intra-arterial tirofiban bolus was given of 2 additional times maximum. Subsequently, the infusion was maintained intravenous at a loading dose of 0.4 μg/kg body weight per minute for 30 min followed by continuous intravenous application at the rate of 0.1 μg/kg body weight per minute for up to 24 h. Recanalization was graded according to modified Treatment in Cerebral Ischemia (mTICI) scale and Arterial Occlusive Lesion (AOL) scale (5). All patients underwent non-enhanced CT immediately after the procedure to ascertain ICH or SAH. Appropriate imaging techniques were used to confirm the presence of ischemic or hemorrhagic complications if patients experienced neurological deterioration at any time within 7 days after the interventional procedure.

### Outcome Measures

The demographic and clinical characteristics, stenting procedures, perioperative management (including antiplatelet resistance test, preoperative antiplatelet agents, dosing of tirofiban for rescue treatment), post-procedural angiographic outcomes, residual stenosis and perioperative complication (complications within 7 days after the procedure including ischemic stroke, symptomatic or asymptomatic intracranial hemorrhage, subarachnoid hemorrhage, systemic bleeding and death) were collected in all patients with AIST. The National Institutes of Health Stroke Scale (NIHSS) were applied at admission, pre-procedure, 24 h post-procedure, 7 days post-procedure. Also neurological variation was assessed by the NIHSS score throughout the hospitalization. Patients' imaging and angiograph were carefully reviewed by one experienced radiologist and one experienced neurointerventionist. Clinical outcomes were assessed by two neurologists.

The efficacy outcome was assessed by the target vessel recanalization in the cerebral angiography and graded using the mTICI scale and AOL scale. Successful recanalization was determined by recanalization with mTICI grade ≥ 2b and AOL 3. Residual stenosis was defined as a diameter of the stenosis > 50% at the end of the intervention. For safety evaluation, any type of intracranial hemorrhage (either symptomatic intracranial hemorrhage or asymptomatic intracranial hemorrhage), subarachnoid hemorrhage (SAH), systemic bleeding, or death were monitored during hospitalization.

### Statistical Analysis

Continuous data are expressed as mean ± SD, while categorical data were presented as numbers and percentages. All statistical analysis were performed using SPSS 19.0 software (SPSS Inc., Chicago, USA).

## Results

During the study period of September 2016 to May 2019, 194 consecutive patients underwent intracranial stent placement for ICAS in our institute. Patient demographic and clinical characteristics are depicted in Table 1. Of these 194 stenting cases, 12 were identified as AIST. The case summaries for these 12 stent procedures are presented in Table 2.

**Table 1:** Baseline demographic and clinical characteristics of patients with ICAS underwent stenting.

**Characteristics *n* = 194**	
Demographics	
Sex, male	142 (73.2%)
Age, years, mean ± SD	60.6 ± 8.9
Medical history	
Hypertension	140 (72.2%)
Diabetes mellitus	86 (44.3%)
Tobacco use	83 (42.8%)
Atrial fibrillation	1 (0.5%)
Lesion location, No. (%)	
Vertebral artery V4 segment (V4)	62 (32.0%)
Basilar artery (BA)	41 (21.1%)
Distal Internal carotid artery (C4–C7)	38 (19.6%)
Middle cerebral artery M1 segment (M1)	53 (27.3%)
Average stenosis	
Degree, %	85.2 ± 7.7%
Stent characteristics	
Stent length, mm	14.2 ± 4.1
Stent diameter, mm	3.27 ± 0.59
Stent types	
Wingspan, No. (%)	75 (38.7%)
Apollo, No. (%)	76 (39.2%)
Neuroform EZ, No. (%)	28 (14.4%)
Other stent, No. (%)	15 (7.7%)

*SD, standard deviation*.

**Table 2:** Baseline and procedural characteristics and outcomes of patients with AIST.

**Characteristics AIST *n* = 12**	
Demographics	
Sex, male	9 (75%)
Age, years, mean ± SD	60.3 ± 8.3
Lesion location, No. (%)	
Vertebral artery V4 segment (V4)	4 (6.5%)
Basilar artery (BA)	3 (7.3%)
Distal Internal carotid artery (C4–C7)	1 (2.6%)
Middle cerebral artery M1 segment (M1)	4 (7.5%)
Average stenosis	
Degree, mean ± SD %	86.1 ± 7.1%
Stent characteristics	
Stent length, mm	14.5± 4.2
Stent diameter, mm	3.2 ± 0.5
Stent types (AIST No. rate)	
Wingspan, No. (%)	8 (10.7%)
Apollo, No. (%)	2 (2.6%)
Neuroform EZ, No. (%)	2 (7.1%)
Time to Thrombus, min, mean ± SD	14.8 ± 6.1
Thrombus disappear, min, mean ± SD	36.5 ± 14.6
Perioperative complication No. (%)	
ICH	0 (0%)
Symptomatic intracranial hemorrhage	0 (0%)
Asymptomatic intracranial hemorrhage	0 (0%)
SAH	0 (0%)
Systemic bleeding	0 (0%)
Ischemic stroke	3 (25%)
Death	0 (0%)
Successful recanalization No. (%)	
mTICI = 3	12 (100%)
AOL = 3	12 (100%)
^a^Residual stenosis No. (%)	0 (0%)

*mTICI, modified Treatment in Cerebral Ischemia; ICH, intracranial hemorrhage (symptomatic or asymptomatic); SAH, subarachnoid hemorrhage*;

a *Residual stenosis, defined as >50% stenosis at the end of the intervention*.

The mean age of treated patients was 60.3 ± 8.3, with the majority (9/12; 75%) of the patients being male. The average stenosis degree was 86.1 ± 7.1%. Of these 12 patients with AIST, 3 events occurred with basilar artery (BA) stent placement, 4 with V4 segment of the vertebral artery (VA) placement, 4 with M1 segment of the middle cerebral artery (MCA) placement, and 1 with ophthalmic internal carotid artery (C6) placement. According to different stents, 8 events occurred during the implantation of Wingspan stents, 2 occurred during the implantation of Apollo stents, 2 events occurred during the implantation of Neuroform EZ stents. The average length and diameter of the stents were 14.5 ± 4.2 and 3.2 ± 0.5 mm.

The overall rate of AIST was 6.2% (12 of 194 patients). The incidences of AIST in MCA, BA, V4, ICA were 7.5, 7.3, 6.5, 2.6%, respectively. AIST developed within 30 min after stent placement in all 12 patients, with 5 grade 1, 6 grade 2, 1 grade 3, the mean time was 14.8 ± 6.1 min after stent deployment.

**Figure 1:**
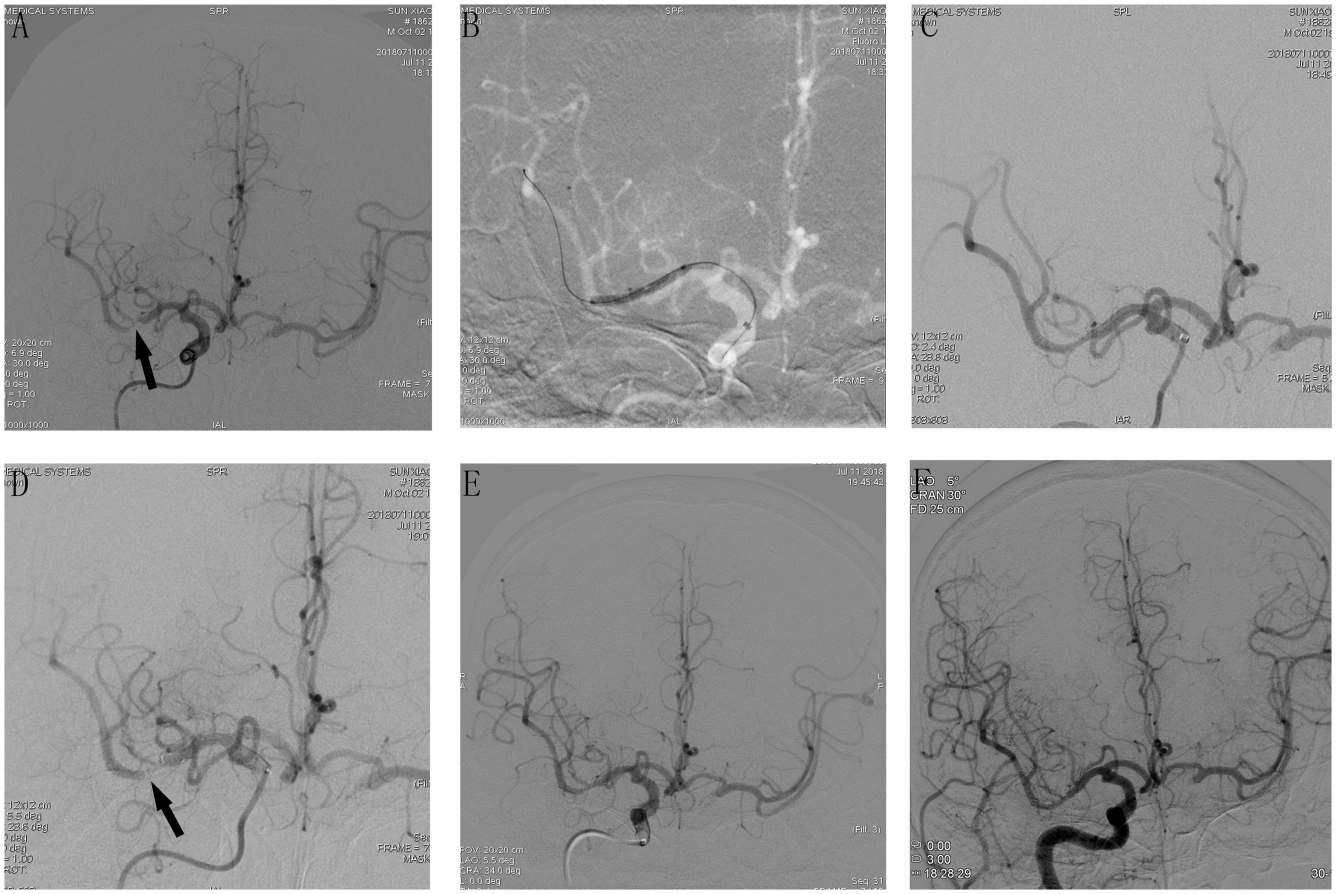
Cerebral angiographic results of patient 11 during the procedure and follow-up. **(A)** Severe stenosis at the M1 segment of the right middle cerebral artery (arrow). **(B)** Angiographic result after predilatation with a conventional balloon. **(C)** Stents release after predilatation, immediate post-placement angiogram demonstrating stent placement in the right MCA with normal flow. **(D)** An additional angiography was done after 10 min, which demonstrated AIST at the M1 segment of the right middle cerebral artery (MCA). **(E)** Intra-arterial bolus followed by intravenous tirofiban was administered and the final angiography 20 min later showed a successful recanalization with mTICI 3 and AOL 3. **(F)** Cerebral angiographic result at the 6-month follow-up with a patent middle cerebral artery.

**Figure 2:**
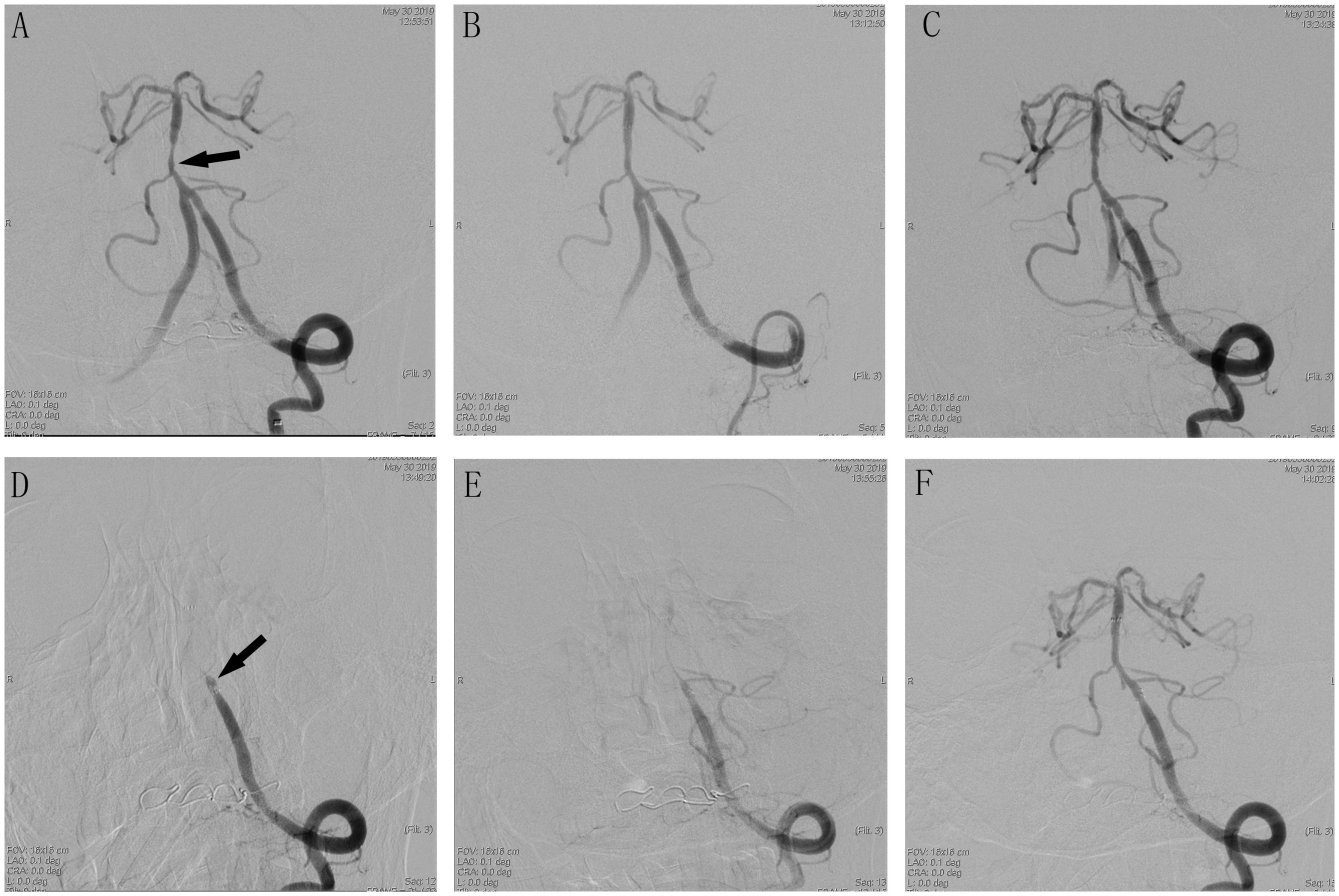
Cerebral angiographic results of patient 12 during the procedure. **(A)** Severe stenosis at the basilar artery (arrow). **(B)** Immediate post-placement angiogram demonstrating stent placement in the vertebral basilar artery with normal flow. **(C)** Visible thrombus in the stent with no flow limiting. **(D)** Delayed angiogram demonstrating complete vertebral basilar artery occlusion. Stent tines are visible superior to the stagnant contrast. **(E)** Visible weak blood flow through the stent after Intra-arterial tirofiban. **(F)** Complete recanalization after treatment with tirofiban.

An overview of all these 12 cases are presented in Table 3. For the 12 patients experiencing AIST, tirofiban as a rescue therapy was administered. Resultantly, successful recanalization was achieved in 12 (100%) patients with mTICI 3 and AOL 3, without any types of ICH, SAH, systemic bleeding, and deaths. Thrombus disappeared from 20 min to 1 h after it was detected, the mean time was 36.5 ± 14.6 min. Nine of these patients were free of ischemic stroke. Three patients suffered post-operative ischemic stroke, of which 1 was major stroke related to perforator occlusion, with the NIHSS score increased from 1 to 8, and 2 were minor stroke without changing in NIHSS score. Perforator occlusion and thromboembolism were thought to be the etiologies of the minor strokes, respectively. Illustrative cases are shown in Figure 1 (patient 11) and Figure 2 (patient 12).

**Table 3:** Characteristics of patients with AIST.

**Patient NO**.	**Admission NIHSS**	**Lesion location**	**Degree of stenosis(%)**	**AA resistance**	**ADP resistance**	**Time to Thrombus, (min)**	**Thrombus grade**	**Thrombus disappear (min)**	**Post-procedure ischemic stroke**	**Post-procedural NIHSS (24 h)**	**Post-Procedural NIHSS (7 days)**	**Stent type**
1	1	C6	80	NO	NO	27	1	55	NO	1	1	W
2	4	V4	80	NO	YES	10	2	55	NO	3	3	W
3	4	V4	98	NO	NO	13	2	31	NO	3	3	W
4	TIA	V4	95	NO	YES	10	1	25	NO	–	–	W
5	1	V4	80	NO	NO	10	2	40	YES	8	8	W
6	9	BA	80	NO	NO	20	1	20	YES	9	9	A
7	5	BA	90	NO	NO	10	2	20	NO	3	3	A
8	TIA	MCA	75	NO	YES	20	2	60	NO	–	–	W
9	1	MCA	85	NO	YES	23	1	30	NO	0	0	W
10	6	MCA	90	NO	YES	10	1	20	NO	5	5	W
11	1	MCA	90	NO	YES	12	2	44	NO	1	1	EZ
12	3	BA	90	NO	NO	12	3	38	YES	3	3	EZ

*TIA, transient ischemic attack; BA, basilar artery; C6, ophthalmic internal carotid artery; MCA, middle cerebral artery; V4, intradural segment of the vertebral artery; W, Wingspan stent; A, Apollo stent; EZ, Neuroform EZ stent*.

## Discussion

The negative result of intracranial stenting in SAMMPRIS (Stenting and Aggressive Medical Management for the Prevention of Recurrent Stroke in Intracranial Stenosis) was primarily due to perioperative complications (14.7%). Of the 21 ischemic events in SAMMPRIS trial, 1 was mixed embolic and perforator territory owing to AIST, 1 had a probable stent thrombosis at 6 days (6). However, this retrospective study was *post hoc*, based on review of available images and clinical data to categorize stroke mechanism. AIST-related ischemic events may be far more than that, actually. Moreover, most previous studies have focused only on post-operative ischemic and hemorrhage complications (7, 8). Based on our findings, it is hypothesized that periprocedural ischemic complications may be caused in part by undetected AIST.

To our knowledge, no one has specifically assessed the safety and efficacy of tirofiban as a rescue agent for AIST during stenting of ICAS on a comparable number of cases. Our study found that the incidence of AIST during intracranial stenting procedures of ICAS was 6.2% in our center and rescue treatment of tirofiban might be sufficient for thrombus resolution and culprit vessel recanalization, without increasing the rate of hemorrhagic complications and death. All of the 12 AIST occurred within 30 min of the stent deployment in our study which was similar to what Lawson had reported during Wingspan intracranial stent placement for ICAS (2). Eight events occurred during the implantation of Wingspan stents. The incidence of AIST is 10.7% of the 75 patients treated with Wingspan stents, which is lower than what Lawson had reported (2). In our center, intraoperative angiography was performed at about 10-min intervals for at least 40 min to ensure timely detection of thrombus formation in the stent or distal end of the targeted artery after stent placement. This is a critical tool for assessing AIST and early diagnosis of AIST is an important condition for complete recanalization.

Recently, risk factors associated with stent thrombosis have been reported in some case series about intracranial stenting and stent-assisted coil embolization of intracranial aneurysms, including the stent design, different stenting procedures, poor stent adherence, smaller diameter of the stent, longer stent length, the location of stenosis, aspirin and/or clopidogrel resistance, and intimal injury from endovascular devices (9–12). In our study, we found a high proportion of antiplatelet resistance in patients who developed AIST. Of the 12 patients with AIST, there were 6 cases (50.0%) with clopidogrel resistance. Antiplatelet resistance likely increased the risk of AIST after intracranial stenting.

Currently, no definite guidelines exist regarding the optimal management of this complication during stent placement of ICAS. The present rescue treatments include intra-arterial thrombolysis with urokinase or tissue plasminogen activator (t-PA), intravenous bolus administration of potent GpIIb/IIIa inhibitors, balloon dilation, and restenting (2, 3, 10). The pathological evaluation of the aspirated thrombus material in clinical and autopsy series has demonstrated that the thrombus is almost totally composed of platelets and contains very low fibrin material (white clot) in the case of AIST, which may impact the efficacy of thrombolysis for this indication (13). GpIIb/IIIa inhibitors may be more effective and safe. As its shorter half-life in plasma and reversible effect on platelet aggregation, tirofiban has fewer bleeding complications than other GpIIb/IIIa inhibitors such as abciximab. And as one of the important mechanism of GpIIb/IIIa inhibitors, tirofiban competitively inhibits platelets GpIIb/IIIa receptor and removes fibrinogen, resulting in disintegration of a hyperacute thrombus (14). Since the binding of the platelet GpIIb/IIIa receptor to fibrinogen is the final pathway leading to platelet aggregation and thrombus formation, tirofiban plays a key role in the process of preventing platelet cross-linking and aggregation and thus prevents thrombus formation by various causes (14–16). In this way, tirofiban can be used as a rescue therapy for AIST. Numerous case reports, case series, retrospective reviews, and meta-analysis have evaluated the safety and efficacy of tirofiban in the treatment of acute thromboembolic complications during stent-assisted coil placement in intracranial aneurysms, with inspiring results (17–20). Additionally, rescue use of tirofiban for acute carotid in-stent thrombosis has been reported for many years with satisfying outcomes (21). The experience with GpIIb/IIIa inhibitors administration for AIST of ICAS is quite limited, with only 2 small case series of abciximab and one small case series of tirofiban to date (1–3). In our series, there were 12 patients experiencing AIST, intra-arterial bolus followed by intravenous tirofiban infusion as a rescue therapy was adopted. Resultantly, successful recanalization was achieved in 12 (100%) patients without any hemorrhagic complications or deaths. What needs to be specially mentioned is that one patient with complete stent occlusions in our series also achieved excellent recanalization. Currently, the standard of care for management of large vessel occlusion strokes is mechanical thrombectomy. However, it is slippery for treatment of AIST-induced vascular occlusion, mechanical thrombectomy may lead to more serious complications due to the stent. We believe that the complete recanalization of stent occlusions in our series was attributed to our close monitoring as well as timely and effective treatment of tirofiban.

The present study has several limitations: (a) this was a non-randomized, retrospective cohort study with its inherent shortcomings; (b) the results obtained from data collected at a single center study with a relatively small sample size and may not be generalizable to other centers for reasons of selection bias; (c) this study contained no control group as all patients suffered from AIST were prescribed tirofiban; and (d) only perioperative result and complications were observed, and the effect needs to be re-evaluated with long-term follow-up. In the future, randomized controlled trials are needed to investigate whether this treatment compares favorably with other management in these patients.

## Conclusions

We observed a non-negligible rate of AIST during intracranial stenting procedures for ICAS. Intra-arterial bolus followed by intravenous tirofiban infusion seems to be efficacious and safe for AIST during stent placement for ICAS, without increasing the rate of hemorrhagic complications and death. However, these findings should be interpreted cautiously because of the aforementioned limitations, and prospective multicenter randomized studies with larger patient number will be required to establish the efficacy and safety of tirofiban.

## Data Availability Statement

The raw data supporting the conclusions of this article will be made available by the authors, without undue reservation, to any qualified researcher.

## Ethics Statement

The studies involving human participants were reviewed and approved by the institutional review board of Shandong Qianfoshan Hospital, Shandong University. The patients/participants provided their written informed consent to participate in this study. Written informed consent was obtained from the individual(s) for the publication of any potentially identifiable images or data included in this article.

## Author Contributions

JH, JT, JiZ, and LS contributed study concepts and design. LS, YS, MZ, WZ, and JuZ organized the database and performed the statistical analysis. LS, HY, YM, and WW wrote manuscript. All authors contributed to manuscript revision, read and approved the submitted version.

## Conflict of Interest

The authors declare that the research was conducted in the absence of any commercial or financial relationships that could be construed as a potential conflict of interest.
